# Application of a water jet for cleaning grease and improving the surface adhesion properties of galvanized steel wire ropes

**DOI:** 10.1038/s41598-022-13955-y

**Published:** 2022-06-11

**Authors:** Xiaojin Miao, Chunlei Zhang, Meiping Wu, Chenglong Ma, Quanlong Wang

**Affiliations:** 1 Jiangsu Key Laboratory of Advanced Food Manufacturing Equipment and Technology, School of Mechanical Engineering, Jiangnan University, Wuxi, 214122 China; 2 Fasten Group Co., Ltd, Wuxi, 214433 China

**Keywords:** Mechanical engineering, Materials science

## Abstract

Traditional cleaning processes may be banned in the near future because of the hazards they pose to the environment. In this study, a water jet was used to clean grease residues from steel wires for the first time. The EDS and SEM results of the steel wire rope surfaces and supplementary water jet impact experiments on galvanized steel plates revealed that when the pressure was lower than 50 MPa and the traverse speed was higher than 600 mm/min, the water jet caused minimal damage to the coating. When the pressure was 5 MPa, the cleaning ratio was between 45 and 60%, and the level of cleaning increased with increasing pressure. Two proposed concepts of exposure ratio and nonexposed area were applied to quantitatively analyze the theoretical upper and lower limits for grease that could be cleaned from two typical structures. The results showed that the lower and upper cleaning limits for structure 7 × 3 were 38.1% and 83.3%, while the lower and upper limits for structure 1 × 3 + 5 × 7 were 35.5% and 59.2%, respectively. This result explains why the grease content of structure 7 × 3 was lower than that of structure 1 × 3 + 5 × 7 after cleaning. In addition, the adhesion test results showed that adhesion to the two kinds of steel wire ropes after cleaning was increased by 126% and 145.71%, respectively, which means that additional processes for improving adhesion could be omitted after using a water jet for cleaning. This is an advantage that traditional cleaning processes do not offer.

## Introduction

Galvanized steel wire ropes are important parts of synchronous belts because of their high strength, good corrosion resistance and good toughness^1^. They play an important role in achieving large capacity and ultralong distance transport. A steel wire rope consists of multiple strands of steel wires wound together. During the process of wire drawing, the wire ropes become contaminated with grease. This residual grease greatly limits the performance of the product^2^. Therefore, the removal of grease is important. At present, factories generally use trichloroethylene to clean grease from the surfaces of steel wire ropes. Trichloroethylene is a commonly used cleaning agent in the industry that offers a short and simple process that is convenient to operate^3^. However, due to the carcinogenicity of trichloroethylene, particularly to the kidney, it has received increasing attention from the International Agency for Research on Cancer and faces the risk of being banned at any time^4,5^. Therefore, to meet the rigorous requirements for environmental protection across the world, it is necessary to seek new steel wire rope cleaning methods.

As a nontraditional cold working technology, water jets have no heat effects, offer high flexibility, are environmentally friendly and are easy to operate^6^. Water is one of the most common energy sources in use. It is nontoxic and harmless and can be reused after filtration. After nearly 50 years of development, this technology and its derivative technologies are increasingly and widely used. The use of abrasive water jets formed by mixing abrasive particles with a water jet has attracted much attention in the fields of milling^7–9^, turning^10,11^, grinding^12^ and polishing^13^. Even so, for achieving its basic function, cleaning, in-depth research valuable and has the potential to expand its fields of application. Water jets peel and wash away dirt to achieve cleaning. Water jet cleaning does not produce a large amount of dust that can pollute the atmospheric environment like simple mechanical cleaning methods do, nor does it produce a large amount of acid and alkali waste liquids that can pollute rivers and soils like chemical cleaning do. As early as the early 1990s, Conn^14^ proposed in-factory applications of water jet cleaning. In recent years, water jet cleaning technology has been extended to a greater number of fields. Careddu et al.^15^ investigated the possible use of water jet technology for graffiti cleaning and determined the best operational conditions for using a water jet machine as a cleaner. Zhang et al.^16^ generated a method for removing coatings from passenger-vehicle plastics based on high-pressure water jet technology to facilitate the recycling of these plastics. Köhler et al.^17^ reported the removal of soil layers from model food by a vertical water jet generated by a solid stream nozzle that moves across a plate, impinging normally on the plate. Fernandes et al.^18^ presented a first-order model for cleaning thin layers of soil materials based on the rate of viscous dissipation in a shallow wedge of material at the cleaning front. Takeuchi et al.^19^ cleaned electrostatic accelerator tubes by applying a high-pressure water jet and examined their high-voltage performances. Shirakawa et al.^20^ researched the efficacy of water jetting for the removal of various types of microbial cells in biofilms. Gabdrakhmanov et al.^21^ analyzed the features of water jet cleaning on a sample of a 3SP welding steel. However, there are no reports on the cleaning of grease from the surfaces of steel wire ropes by water jets. Previous studies have focused on simple structures such as plane, quasi plane or circular surfaces. These structures do not greatly obstruct the water jet cleaning process. However, the structure of steel wire ropes is quite complex. Different stranding methods form different structures, which makes it difficult to clean the surfaces inside the steel wire ropes using a water jet. Therefore, the influence of structure on cleaning efficacy is one of the main differences between cleaning steel wire ropes and cleaning other structures.

In addition, in recent years, many experts, such as Srivastava^22^, Muruganandhan^23^, Ijiri^24^, and Lehocká^25^, have proposed that water jets can be used for peening metals to improve surface performance. Their research consistently shows that the impact from a water jet is an effective way to improve the surface performance of metals. This means that during the cleaning of grease, the water jet also affects surface performance. Preliminary experimental results show that surface adhesion is improved after water jet cleaning. Surface adhesion is very important for steel wire ropes. It is a parameter that must be tested before delivering each batch of steel wire rope. The improvement in adhesion brought about by the process of grease cleaning can eliminate the subsequent adhesion improvement processes that must be typically carried out, which simplifies process flow and reduces costs. From this point of view, a water jet cleaning process has additional advantages over traditional cleaning processes.

Therefore, this study selected two kinds of steel wire ropes with typical structures to study the process of water jet cleaning on steel wire ropes on the premise that the zinc coating would not be greatly damaged. The underlying factors driving the influence of structure on cleaning were quantitatively analyzed, and the effect of water jet cleaning on the improvement in surface adhesion of the steel wire ropes was studied. The results of this work can help promote the application of water jets in the field of steel wire rope cleaning.

## Materials and methods

**Figure 1:**
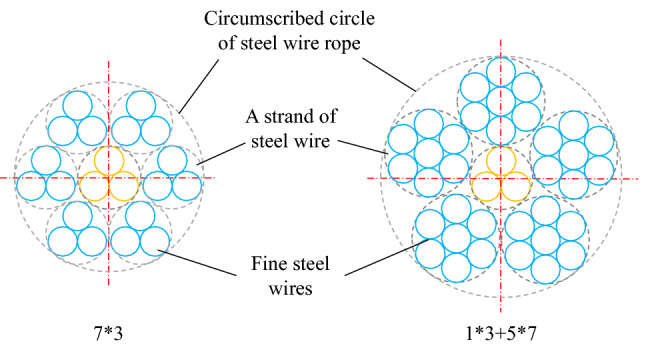
Two structures.

The galvanized steel wire ropes used in the experiments were provided by Fasten Group Co., Ltd. This company produces steel wire ropes. It is at the forefront of the industry and is a worldwide leader in the production of steel wire ropes. To study the factors affecting the cleaning efficiency of the water jet, two kinds of steel wire ropes with different structures were selected for the experiments. The structures are shown in Fig. 1. The grease originated from the heavy-duty gear oil produced by FAW. The chemical composition of the steel wire is shown in Table 1, and the parameters of the steel wire ropes are shown in Table 2.

**Table 1:** Chemical composition of the steel wire.

Element	C	Si	Mn	P	S	Fe	Others
Content (wt%)	0.72	0.19	0.52	0.009	0.005	97.73	0.826

**Table 2:** Parameters of steel wire ropes.

Samples	Structures	Number of fine steel wire	Diameter of circumscribed circle (mm)	Thickness of zinc coating (μm)	Grease content
1	7 × 3	21	0.91	≤ 50	Moderate
2	1 × 3 + 5 × 7	38	1.21	≤ 50	Moderate
3	7 × 3	21	0.91	≤ 50	Heavy
4	1 × 3 + 5 × 7	38	1.21	≤ 50	Heavy

A hot-dip galvanizing process was used, and the coating thickness did not exceed 50 μm. The steel wire rope with a structure of 7 × 3 was wound with 7 strands of steel wires, and each strand of steel wire was wound with 3 fine steel wires. The circumscribed circle diameter of this structure was 0.91 mm. The steel wire rope with a structure of 1 × 3 + 5 × 7 was wound with 6 strands of steel wires. The strand of steel wire in the middle was wound with 3 fine steel wires, and the other 5 strands of steel wires were wound with 7 fine steel wires. The circumscribed circle diameter of the steel wire rope of this structure was 1.21 mm. The initial content of grease was usually stable, but a large grease content occasionally occurred due to various factors. Therefore, two cases, moderate grease and heavy grease, were studied.

An important premise for using water jets for cleaning steel wire ropes is that the water jets will not cause substantial damage to the zinc coating. This is also an important basis for evaluating the selection of process parameters for the water jet. A five-axis ultrahigh pressure water jet machine tool, DWJ3020-BB-X5, was used to carry out the water jet experiments that were conducted on the steel wire ropes after ultrasonic cleaning. The maximum pressure and flow of the machine tool could reach 413.7 MPa (60,000 psi) and 3.6 L/min. The steel wire ropes before and after water jet impact were analyzed by a Sigma HD scanning electron microscope, 51-XMX1003 energy dispersive X-ray spectrometer and a LEICA-DM-2700M optical microscope. Additional experiments were carried out on galvanized steel plates for better observation and analysis. The influence of the water jet on the zinc coating was analyzed by adjusting the process parameters, and this was used as the basis for determining the process parameters for follow-up studies. The preliminary settings for the process parameters of the water jet are shown in Table 3.

**Table 3:** Preliminary settings for process parameters.

Process parameters	Values
Pressure (MPa)	50, 100, 150, 200
Traverse speed (mm/min)	600, 1200
Standoff distance (mm)	175

The standoff distance was set to the maximum value allowed by the equipment to improve the cleaning coverage as much as possible. This work focused on grease cleaning efficiency. The selection principle for the process parameters was that the zinc coating was not damaged. Each sample in Table 1 had 9 sections, and the length of each section was 30 cm. The experiment was divided into 3 groups (12 groups in total). Each group was tested 3 times, and the average value was taken for analysis. The steel wire ropes of the first two groups were first cleaned by a water jet at different pressures. Then, they were placed in an ultrasonic cleaning machine for 30 min. An appropriate amount of pyruvate was placed in the ultrasonic cleaning machine. The steel wire ropes of Group 3 were directly placed in the ultrasonic cleaning machine for 30 min without water jet cleaning.

Measuring the water contact angle and weighing are the usual methods used to evaluate the cleaning efficiency for grease. It is very difficult to measure the water contact angle of steel wire ropes due to the small diameter and the cylindrical surface. Moreover, water contact angle analysis cannot quantitatively indicate the cleaning efficiency but can only indirectly reflect the cleanliness of the surface. Therefore, weighing was the method used in this study. During this research, the steel wire ropes were weighed during three stages, namely, before each experiment, after water jet cleaning and after ultrasonic cleaning.

The weight of the steel wire rope before water jet cleaning was recorded as ${W}_{0\_ij}$, the weight after water jet cleaning was recorded as ${W}_{1\_ij}$, and the weight after ultrasonic cleaning was recorded as ${W}_{2\_ij}$. $i$ is the $i$-th type of steel wire rope, and $j$ is the $j$-th group of experiments.

In the third group of experiments, the steel wire ropes were not cleaned by a water jet, so the weight loss after ultrasonic cleaning is approximately the total amount of grease.

Thus,

1 $${{{W}_{Ti}=W}_{0\_i3}-W}_{2\_i3},$$

where ${\mathrm{W}}_{\mathrm{Ti}}$ is the total grease on the $\mathrm{i}$-th type of steel wire rope.

The grease content after water jet cleaning is

2 $${\eta }_{ij}=\frac{{W}_{1\_ij}-{W}_{2\_ij}}{{W}_{1\_ij}},$$

where ${\eta }_{ij}$ is the grease content of the $i$-th type of steel wire rope after the $j$-th group of experiments.

The cleaning ratio of the water jet is

3 $${\lambda }_{ij}=\frac{{W}_{0\_ij}-{W}_{1\_ij}}{{W}_{Ti}}=\frac{{W}_{0\_ij}-{W}_{1\_ij}}{{W}_{0\_i3}-{W}_{2\_i3}},$$

where ${\lambda }_{ij}$ is the cleaning ratio of the $i$-th type of steel wire rope after the $j$-th group of experiments.

In addition, this study also investigated the improvement in surface adhesion after the use of the water jet. Steel wire ropes with the two above structures were sampled. The length of the steel wire rope was 30 m, which could be tested 4 times at 4 positions by an adhesion tester from Fasten Group Co., Ltd.

## Results and discussion

### Damage to the zinc coating

**Figure 2:**
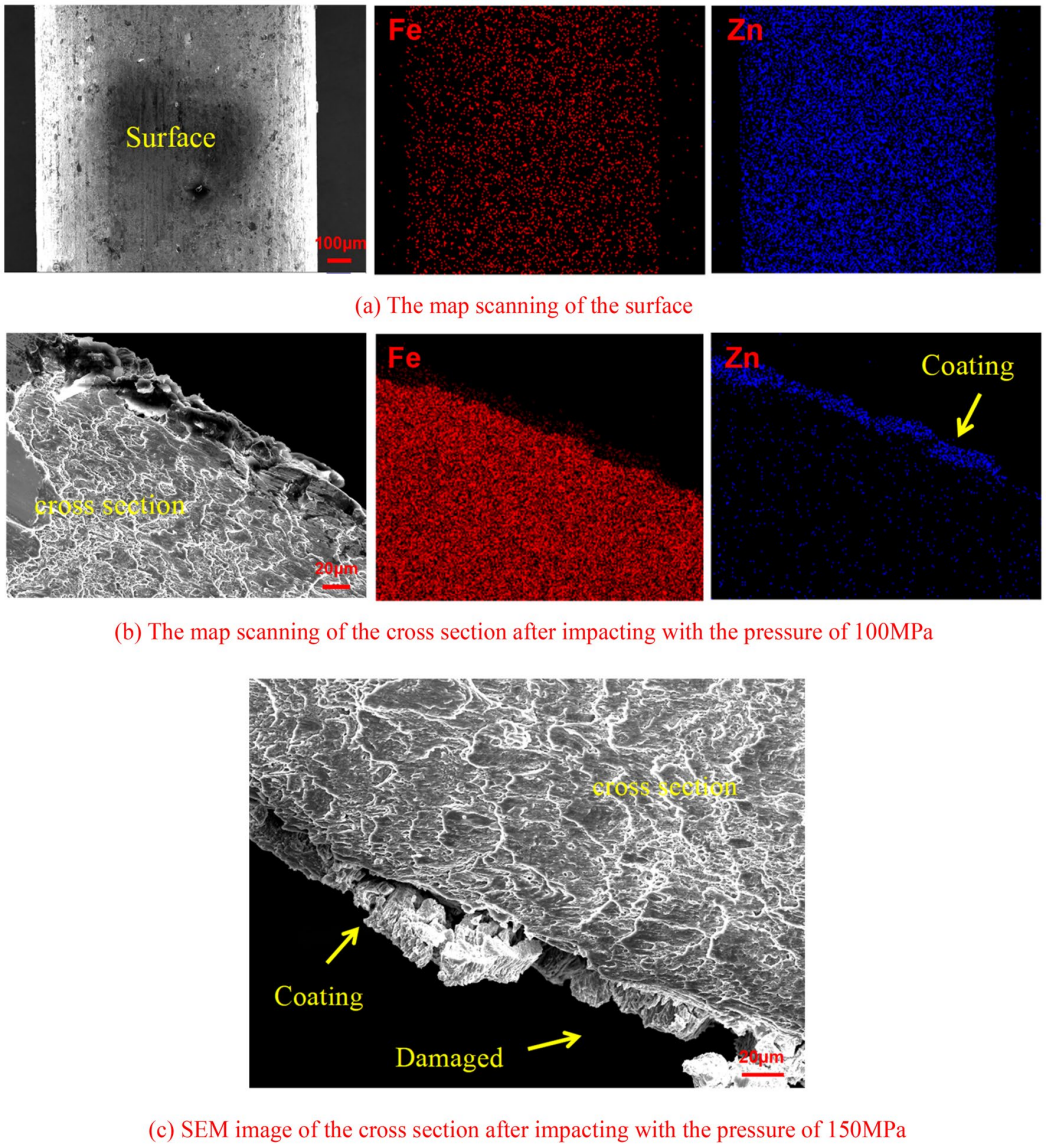
SEM and EDS results.

Water jet impact experiments were carried out on the steel wire ropes after ultrasonic cleaning. Therefore, the grease on the surface was cleaned before the experiment. The surface of the steel wire was galvanized. The zinc coating is prone to oxidation reactions when it is exposed to air^26,27^. Therefore, the outermost layer of the steel wire rope is ZnO. Figure 2a shows the elemental distribution on the surface of the steel wire. There was a large amount of Fe in the coating. This Fe diffused into the coating during hot-dip galvanization^28,29^. The distribution of Fe and Zn on the steel wire surface was uniform. The elemental analysis results of the steel wire surface are shown in Table 4. The O/Zn atomic ratio of the surface without water jet impact was nearly 1:1. The zinc oxide layer did not appear to have been damaged. The water jet impact experiment was carried out at a pressure of 100 MPa and a traverse speed of 1200 mm/min. The coating after water jet impact did not appear to be significantly damaged, and the coating maintained good integrity, as shown in Fig. 2b. However, through elemental analysis of the steel wire surface, it was found that the proportion of Zn increased after impacting. This means that the zinc oxide layer was damaged by water jet impact. Under this condition, the water jet caused slight damage to the coating. The pressure was increased to 150 MPa and an abrasive water jet cleaning experiment was carried out. The results show that the water jet caused substantial damage to the coating. The coating in the impact area was almost completely damaged, as shown in Fig. 2c.

**Table 4:** Elemental analysis of the surface.

Element	Before impacting		After impacting	
	Weight %	Atomic %	Weight %	Atomic %
O	18.46	47.75	16.51	44.38
Fe	5.89	4.37	6.06	4.67
Zn	75.65	47.89	77.44	50.96

**Figure 3:**
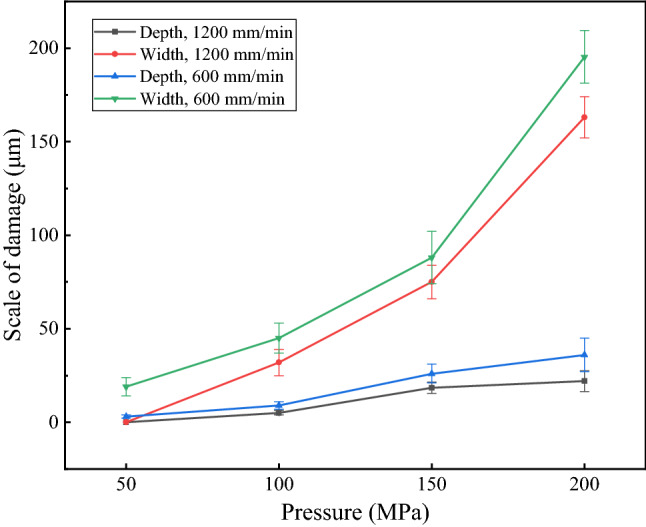
Scale of damage after impact.

The cylindrical shape and the small diameter of the steel wire introduced substantial challenges to in-depth analysis. Thus, supplementary experiments were carried out. The use of a galvanized steel plate instead of steel wire rope to carry out the water jet impact experiments was more conducive to observation and analysis. Since the galvanization process and steel composition were the same, this replacement would not fundamentally affect the conclusions. The thickness of the coating of the steel plate was approximately 50 μm. Water jet impact experiments were carried out on the galvanized steel plate, and the results are shown in Fig. 3.

Figure 3 shows that when the traverse speed was 1200 mm/min, the water jet at a pressure of 50 MPa caused minimal damage to the coating. However, when the pressure reached 100 MPa, the zinc coating was slightly damaged. When the pressure was 150 MPa, the damage was substantial. The depth of the damaged area was approximately 19 μm. With a further increase in pressure to 200 MPa, the depth of the damaged area reached 22 μm. However, the rate of increase of the depth decreased. Theoretically, with increasing pressure, the energy growth rate of the water jet was fixed. However, due to the increase in the real-time standoff distance, the energy of the water jet at the axis decreased, which affected the effective erosion energy of the water jet on the coating^30^. Both the depth of the damaged area and the width increase with increasing pressure. This phenomenon is related to the energy distribution characteristics of the water jet. The energy of the water jet decays gradually from the axis to the periphery^31^. Such an energy distribution characteristic means that when the overall energy is low, only a small area near the axis can cause damage to the material. As the overall energy increases, the area that can cause damage increases. The rate of increase of the width increased. This is because the increase in the real-time standoff distance expands the effective contact area between the water jet and the coating. The same trend was shown at the traverse speed of 600 mm/min. However, the depth and width of the damaged area were greater. A low traverse speed corresponds to a large effective impact energy^32^. In addition, when the pressure was 50 MPa and the traverse speed was 600 mm/min, the impact caused slight damage to the coating. This means that to ensure the coating was minimally damaged, the pressure had to be less than 50 MPa, and the traverse speed had to be more than 600 mm/min. A high traverse speed is more advantageous because it achieves high cleaning efficiency. Efficiency is crucial to factory applications.

### Cleaning efficiency

Grease content is a very important parameter for steel wire ropes. The qualified grease content of steel wire ropes should be less than 0.15%. Water jet cleaning experiments were carried out on steel wire ropes with different structures and different grease conditions. To ensure that the zinc coating was not damaged, the pressures were set to 5 and 50 MPa according to the analysis in the previous section. The traverse speed was 1200 mm/min.

**Figure 4:**
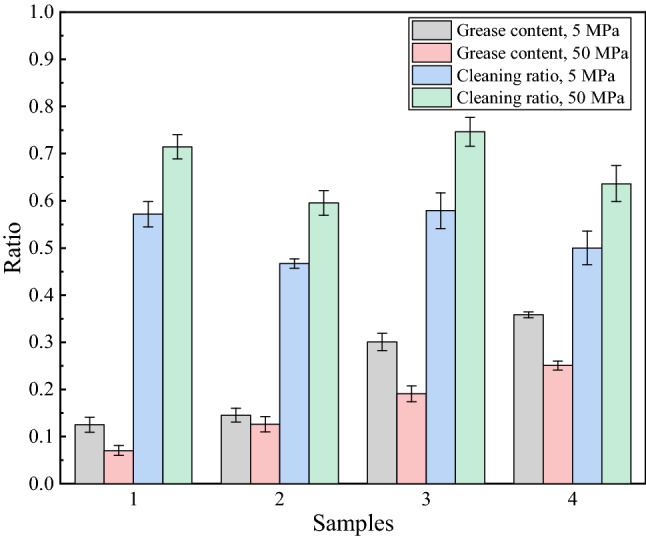
Cleaning ratio of the water jet and grease content after cleaning.

Figure 4 shows the cleaning ratio of the water jet and grease content of the different steel wire ropes after water jet cleaning under different pressures. The data in the figure were calculated according to Eqs. (1)–(3). The figure shows that the water jet had a good cleaning effect on the steel wire ropes with moderate amounts of grease. The grease content after water jet cleaning met the quality requirements. However, for steel wire ropes containing heavy amounts of grease, the grease content after water jet cleaning did not meet the requirements. In addition, the grease content after water jet cleaning under a pressure of 50 MPa was lower than that under a pressure of 5 MPa. Each group of experiments showed this trend. Pressure had an impact on the cleaning of grease. High pressure corresponded to a great impact, which was more conducive to the cleaning of grease.

The ratio of the weight of the grease cleaned by the water jet to the total was taken as the cleaning ratio of the water jet to better analyze the cleaning efficiency. As shown in Fig. 6, when the pressure was 5 MPa, the cleaning ratio of the water jet was between 45 and 60%, but when the pressure was 50 MPa, the cleaning ratio increased slightly. This is also because the higher the pressure is, the stronger the impact of the water jet. It should be noted that the cleaning ratio of the water jet was stable, regardless of whether the grease content was moderate or heavy. The steel wire ropes with heavy grease contents had large amounts of grease residues after cleaning, which is why these steel wire ropes could not meet the grease content requirements after cleaning by a water jet.

**Figure 5:**
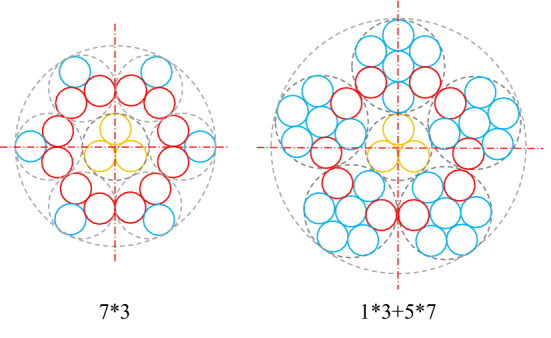
The minimum exposure ratio.

The structure of samples 1 and 3 is the so-called structure 7 × 3, while that of samples 2 and 4 is the so-called structure 1 × 3 + 5 × 7. By analyzing the data in Fig. 5, it was found that the surface grease cleaning ratio of the steel wire ropes of structure 1 × 3 + 5 × 7 by the water jet was generally lower than that of structure 7 × 3. This is closely related to the cleaning characteristics of the water jet. The water jet removes grease through the impact from water. However, a steel wire rope consists of multiple fine steel wires. Water can only clean the exposed surfaces of steel wire ropes. If the structure is different, the exposed surface changes. Therefore, the concepts of exposure ratio and nonexposed area were proposed to analyze the influence of structure on the cleaning efficiency.

The exposure ratio is the ratio of the exposed surface to the total surface. It reflects the lower limit of the surface that can be cleaned. Since a steel wire rope is wound by multiple strands of fine steel wires, the position of the fine steel wires at each cross section is different. Therefore, it is necessary to analyze the minimum exposure ratio of the two kinds of steel wire ropes. As shown in Fig. 5, when the steel wires marked in red are close to each other, the internal area is nearly isolated. In this position, the exposure ratio is the lowest. The minimum exposure ratios of the structures are as follows:

4 $${\mu }_{0.91}=\frac{\left(\frac{1}{3}\times 2+\frac{2}{3}\right)\times 6}{6\times 3+3}\times 100\%=38.1\%,$$

5 $${\mu }_{1.21}=\frac{\left(\frac{126^\circ }{360^\circ }\times 2+\frac{2}{3}\times 3\right)\times 5}{5\times 7+3}\times 100\%=35.5\%,$$

where ${\mu }_{0.91}$ is the minimum exposure ratio of structure 7 × 3 and ${\mu }_{1.21}$ is the minimum exposure ratio of structure 1 × 3 + 5 × 7.

It can be seen from Eqs. (4) and (5) that the minimum exposure ratio of structure 7 × 3 is larger than that of structure 1 × 3 + 5 × 7, which means that its lower cleaning limit is higher.

**Figure 6:**
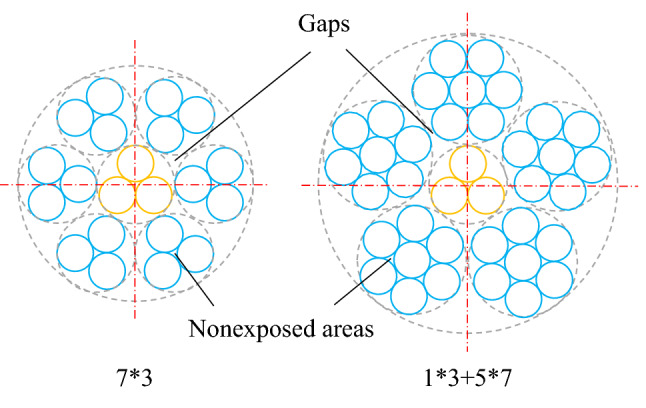
Gaps and nonexposed areas.

At other cross sections, there are gaps between the strands of wires. High-pressure water can clean the fine steel wires inside the steel wire rope through the gaps. However, there are nonexposed areas within each steel wire rope. Such an area is formed by three fine steel wires closely wound together, as shown in Fig. 6. Clearly, the grease in the nonexposed areas is difficult to clean with a water jet. Therefore, it can closely reflect the upper cleaning limit.

The proportions of nonexposed areas of the two structures are as follows:

6 $${\sigma }_{0.91}=\frac{\frac{1}{6}\times 3\times 7}{6\times 3+3}\times 100\%=16.7\%,$$

7 $${\sigma }_{1.21}=\frac{\frac{1}{6}\times 3\times 6\times 5+\frac{1}{6}\times 3}{5\times 7+3}\times 100\%=40.8\%,$$

where ${\sigma }_{0.91}$ is the proportion of nonexposed areas of structure 7 × 3 and ${\sigma }_{1.21}$ is the proportion of nonexposed areas of structure 1 × 3 + 5 × 7.

According to Eqs. (6) and (7), the proportion of the nonexposed area of structure 7 × 3 is much lower than that of structure 1 × 3 + 5 × 7. The upper cleaning limits of the two steel wire ropes are 83.3% and 59.2%, respectively.

Based on the above analysis, the structure 7 × 3 corresponds to a larger minimum exposure ratio and has a smaller proportion of nonexposed areas. Therefore, a steel wire rope of this structure can be cleaned more thoroughly. This explains why the water jet cleaning ratio corresponding to this structure is higher.

**Figure 7:**
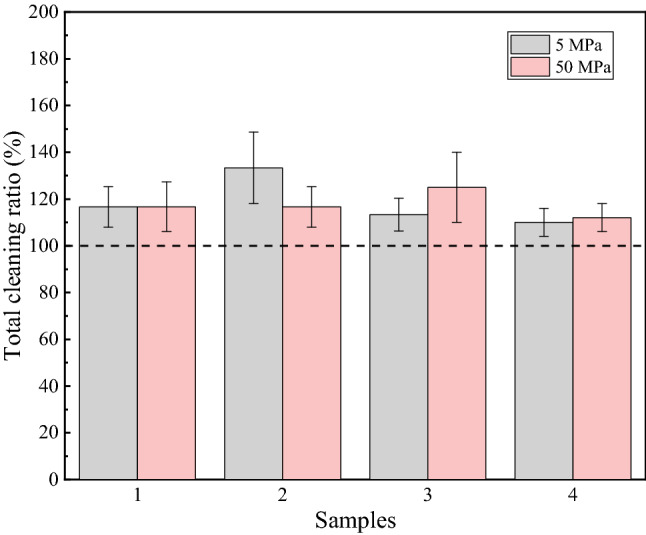
Cleaning ratio.

**Figure 8:**
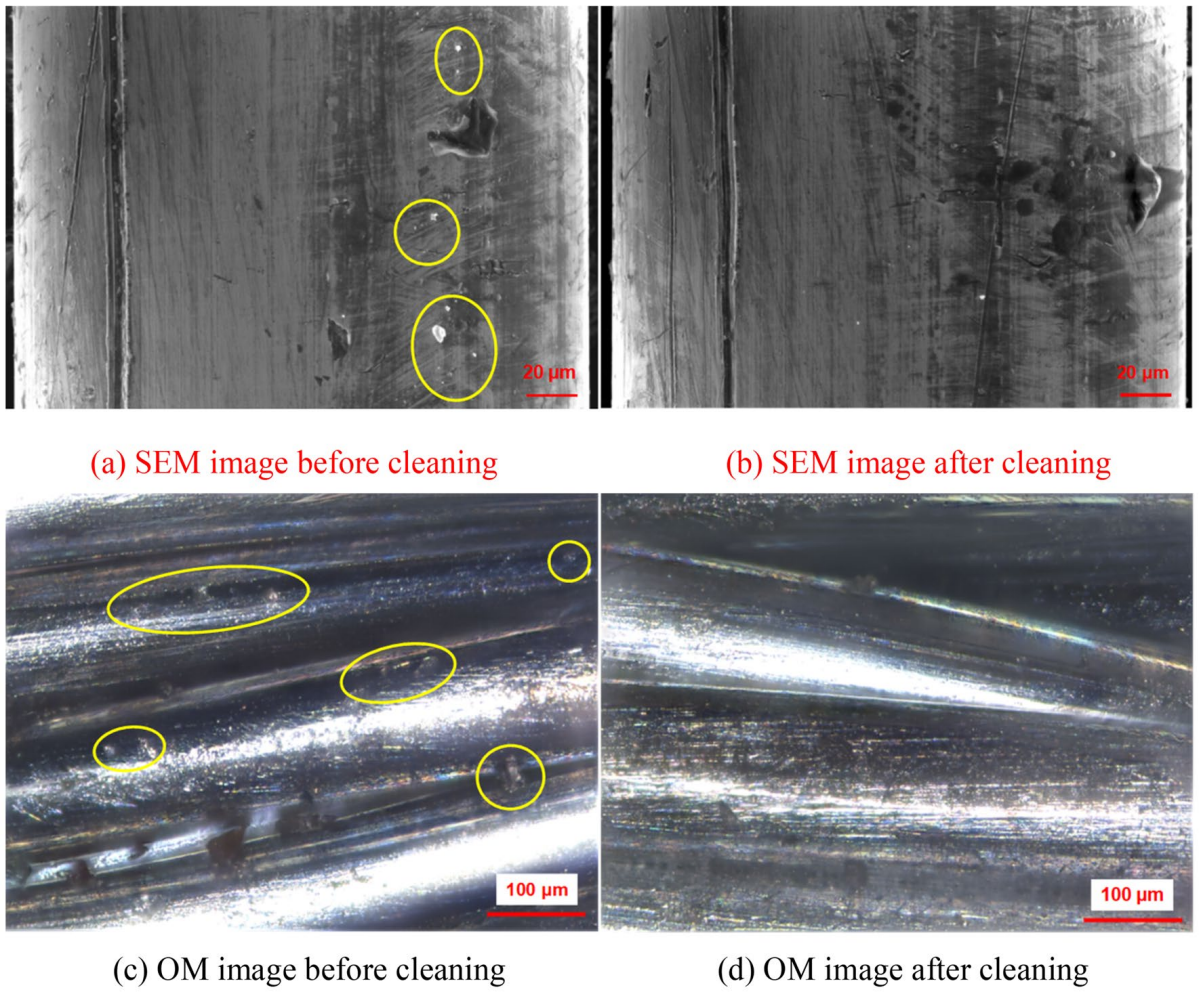
Enlarged view of the surfaces of the steel wire ropes (P = 50 MPa, U = 1200 mm/min).

The steel wire ropes were cleaned by a water jet and ultrasonication. Ideally, the sum of the reduced weight after cleaning should be equal to the total grease weight. However, through the data in Fig. 7, it was found that the sum of the reduced weight was generally larger. This means that the water jet also removed substances other than grease. According to the previous analysis, when the pressure was 50 MPa, the water jet did not damage the coating. When the pressure was dropped to 5 MPa, the water jet did not damage the coating. It can be inferred that if the coatings were damaged, there would be large gaps between the damage that occurred at the two different pressures. However, Fig. 7 shows that the cleaning ratios under the two pressures were at the same level, indicating that the water jet removed substances other than the zinc coating. As shown in Fig. 8a,c, some zinc slag is present on the steel wire ropes. After water jet cleaning, the zinc slag was effectively removed, and the surface quality was significantly improved, as shown in Fig. 8b,d. The water jet can cleaned the zinc slag. This is why the total weight reduced by cleaning was higher than the weight of grease.

### Surface adhesion

**Figure 9:**
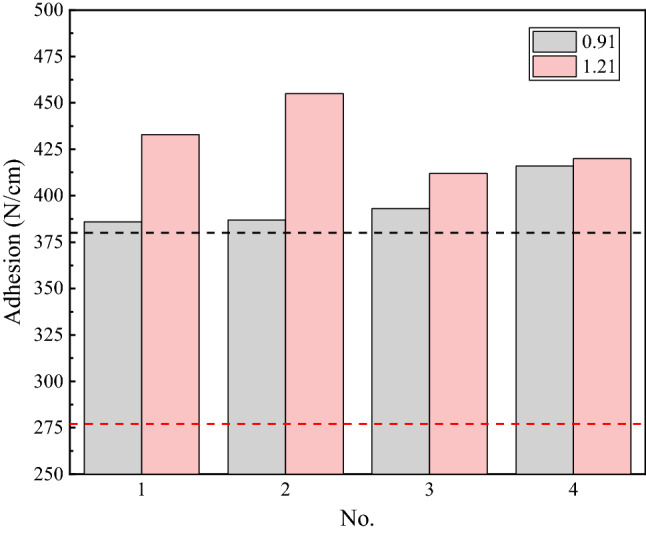
Adhesion of the surfaces cleaned by a water jet (P = 50 MPa, U = 1200 mm/min).

Peening by a continuous water jet can provide compressive stress and induce microstructural evolution^33^. Therefore, after water jet cleaning, the performance of the surface of the steel wire rope changed. Steel wire rope surface adhesion was tested, and the results are shown in Fig. 9.

The factory-required value for the surface adhesion of structure 7 × 3 is 380 N/cm, while the value of structure 1 × 3 + 5 × 7 is 277 N/cm. Figure 9 shows that the steel wire ropes cleaned by a water jet met the requirements for surface adhesion. The average surface adhesion of the steel wire ropes cleaned by traditional cleaning methods is only approximately 175 N/cm. The average surface adhesion of structure 7 × 3 cleaned by a water jet was 395.5 N/cm, which is 126% higher than the traditional value and 4.08% higher than the required value. The average surface adhesion of structure 1 × 3 + 5 × 7 is 430 N/cm, which is 145.71% higher than the traditional value and 55.23% higher than the required value.

The water jet improved adhesion by peening the surface of the steel wire ropes. This means that the larger the exposed surface area is, the more significant the improvement by the water jet on adhesion. The exposed surface areas of the two structures were as follows:

8 $${S}_{0.91}=\left(\frac{1}{3}\times 2+\frac{2}{3}\right)\times 6\times \pi dl=8\pi dl,$$

9 $${S}_{1.21}=\left(\frac{126^\circ }{360^\circ }\times 2+\frac{2}{3}\times 3\right)\times 5\times \pi dl=13.5\pi dl,$$

where ${S}_{0.91}$ is the exposed surface area of structure 7 × 3, ${S}_{1.21}$ is the exposed surface area of structure 1 × 3 + 5 × 7, $d$ is the diameter of the fine steel wire, and $l$ is the length of the fine steel wire.

Equations (8) and (9) show that although the proportion of nonexposed areas of structure 1 × 3 + 5 × 7 was lower than that of structure 7 × 3, the total exposed surface area was 68.8% higher than that of structure 7 × 3. Therefore, the surface adhesion of structure 1 × 3 + 5 × 7 was higher than that of structure 7 × 3.

## Conclusions

In this work, the use of a water jet was proposed for cleaning steel wire ropes. It was found that a water jet can be used to clean steel wire ropes with moderate levels of grease. This process is not only environmentally friendly but can also offer savings on cleaning costs and shorten the process flow. When the pressure was lower than 50 MPa and the traverse speed was more than 1200 mm/min, the water jet caused minimal damage to the coating. The concepts of exposure ratio and nonexposed area were proposed to analyze the influence of the structures of the steel wire ropes on water jet cleaning efficiency. The minimum exposure ratio of structure 7 × 3 was 38.1%, which was 2.6% higher than that of structure 1 × 3 + 5 × 7. Moreover, the nonexposed area of structure 7 × 3 was 16.7%, which was 24.3% lower than that of structure 1 × 3 + 5 × 7. The upper and lower cleaned limits for structure 7 × 3 were both higher than those of structure 1 × 3 + 5 × 7, so the water jet showed a higher cleaning ratio for the steel wire ropes with this structure. In addition, cleaning of grease and zinc slag also improved surface adhesion. The results of the surface adhesion tests showed that the average surface adhesion of structure 7 × 3 was 395.5 N/cm, which was 126% higher than the traditional value and 4.08% higher than the factory-required value. The average surface adhesion of structure 1 × 3 + 5 × 7 was 430 N/cm, which was 145.71% higher than the traditional value and 55.23% higher than the factory-required value. Although the exposure ratio of structure 1 × 3 + 5 × 7 was low, the total exposed surface area was 68.8% higher than that of structure 7 × 3. This result explains why the surface adhesion of this structure was improved more significantly.

## Data Availability

The data will be available from the corresponding author on reasonable request.
